# Time trend and Bayesian mapping of multiple myeloma incidence in Sardinia, Italy

**DOI:** 10.1038/s41598-022-06745-z

**Published:** 2022-02-17

**Authors:** Giorgio Broccia, Jonathan Carter, Cansu Ozsin-Ozler, Federico Meloni, Ilaria Pilia, Sara De Matteis, Pierluigi Cocco

**Affiliations:** 1 Department of Haematology and Bone Marrow Transplants, Hospital A. Businco, 09121 Cagliari, Italy; 2 University of Coventry, Coventry, CV1 5FB UK; 3 Department of Paediatric Dentistry, Hacettepe University, Faculty of Dentistry, Ankara, Turkey; 4 Department of Medical Sciences and Public Health, University of Cagliari, 09042 Monserrato, Italy; 5 Division of Population Health, Centre for Occupational and Environmental Health, University of Manchester, Manchester, UK

**Keywords:** Oncology, Risk factors

## Abstract

A few reports have described increasing trends and spatial distribution of multiple myeloma (MM). We used a validated database including the 1606 cases of MM diagnosed in Sardinia in 1974–2003 to explore its time trend, and we applied Bayesian methods to plot MM probability by administrative unit on the regional map. Over the 30 years of observation, the MM standardized incidence rate (standard world population, all ages) was 2.17 × 10^–5^ (95% CI 2.01–2.34), 2.29 (95% CI 2.06–2.52) among men, and 2.06 (95% CI 1.83–2.28) among women. MM incidence increased by 3.3%/year in 1974–2003, in both males and females, particularly among the elderly and in the high incidence areas. Areas at risk tended to cluster in the north-eastern part of the region. A higher proportion of elderly in the resident population, but not socioeconomic factors, nor livestock farming, was associated with higher incidence rates. The steep upward time trend and the spatial clustering of MM suggest interactions between genetic and environmental determinants that might be more efficiently investigated in the areas at risk.

## Introduction

Multiple myeloma (MM) is a mature B cell lymphoma, which worldwide standardized rate is 2.1 × 10^–5^ (95% CI 1.8–2.3)^1^, ranging from 0.1 to 4.5 among the male population of Asian countries to 10.2–13.1 × 10^–5^ among the male Afro-American population of Texas and Wisconsin^2^. MM incidence is more elevated among men; increases sharply with age^3^; and it occurs more frequently among several occupations, including farmers, cleaners, printers, painters, food processors, and teachers, in several industry jobs, and following exposure to ionizing radiation, certain pesticides, and solvents^4–10^. However, its aetiology is still unclear.

As it concerns its time and space variability, an increasing time trend of MM has been described in the Czech Republic^11^, South America^12^, Taiwan^13^, and in Canada^14^. Three studies described an excess risk in metropolitan and urban areas^12,14,15^, while no spatial clustering of MM cases was observed in a UK study^16^.

The Sardinian population is well known for its peculiar genetic features^17^, which have provided the ideal setting for studies on the genes associated with the local high prevalence of male centenarians^18^, multiple sclerosis^19^, or type I childhood diabetes. The study of spatial distribution of MM incidence over the island might provide clues for future more in-depth studies on genetic susceptibility and gene-environment interactions in MM aetiology.

Based on the data reported in the 11th Edition of the International Agency for Research on Cancer (IARC) Cancer Incidence in the Five Continents (CI5-11), the incidence rate ranges 2.6–5.4 × 10^–5^ across the 33 Italian Cancer Registries^2^. The history of cancer registration in Sardinia is relatively recent, fragmented, and discontinuing: the Sassari Cancer Registry, covering the northern part of the island and about 30% of the resident population, started in 1993 and ceased operating in 2013; the Nuoro Cancer Registry, covering the central-eastern part of the island and 13% of the total population, started in 2003 and it keeps functioning. After several decades of unsuccessful attempts, a Cancer Registry has been planned for southern Sardinia, but it might take an unpredictable length of time to start operating regularly. The standardized incidence (world population, all ages) reported in the CI5-11 is 2.9 among males and 2.8 × 10^–5^ among females in northern Sardinia, and 4.0 among males and 2.1 among females in its central-eastern part^2^.

To get around the lack of an official system of regular surveillance of cancer incidence, in 1974, the chief haematologist of the Cagliari Oncology Hospital (BG) initiated a database of incident cases of haemolymphatic malignancies over the whole region, with the collaboration of all the clinical, surgical and pathology departments, social security agencies, and health authorities, and he kept updating it up to 2003, as described elsewhere^20^. To explore the time trend and the geographical distribution of multiple myeloma over the territory of the island, we used such database, which was previously validated by comparison with mortality and hospitalization data^21^.

## Results

### Time trend in MM incidence

**Figure 1:**
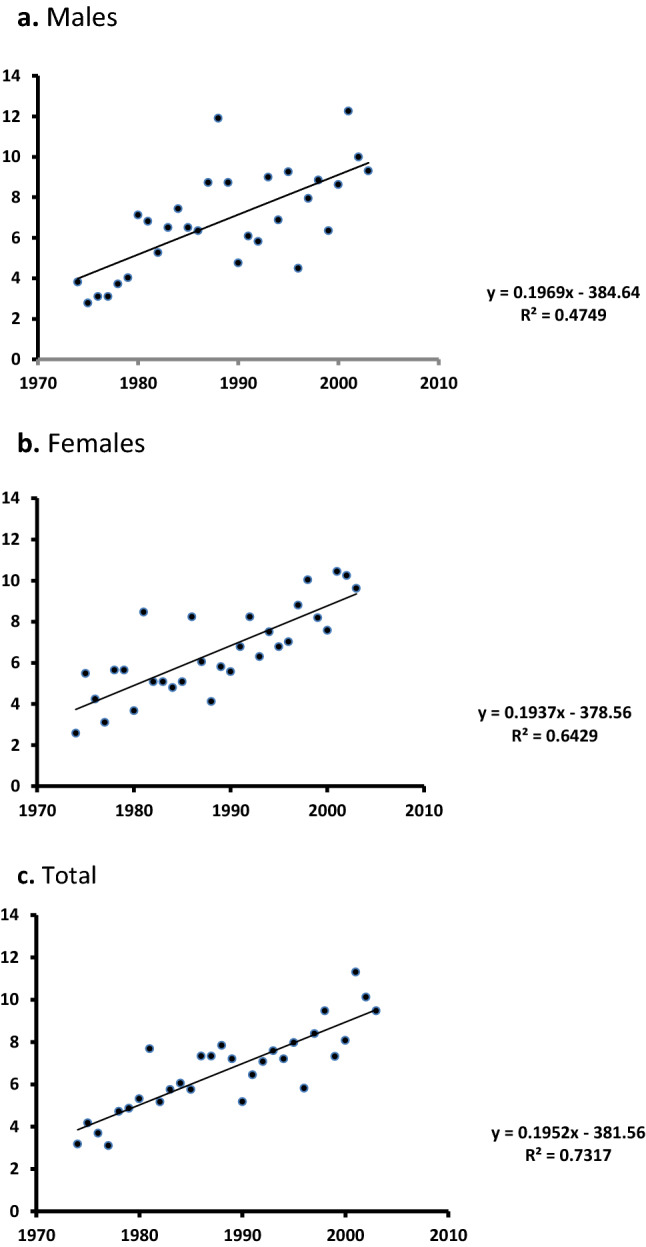
1974–2003 annual incidence rate of multiple myeloma in Sardinia, Italy, by gender (**a** males, **b** females) and overall (**c**).

In 1974–2003, 1606 MM cases occurred among the Sardinian population. Based on the standard World population, the incidence rate over these three decades was 2.17 × 10^–5^ (95% CI 2.01–2.34) for the total population (all ages), 2.29 (95% CI 2.06–2.52) among males, and 2.06 (95% CI 1.83–2.28) among females, with a male/female ratio of 0.95. The crude rate over the study period was 2.73 × 10^–5^ (95% CI 2.57–2.89) among the total population, 3.17 (95% CI 2.94–3.40) among males, and 2.30 (95% CI 2.07–2.53) among females. With Poisson regression analysis, the average annual increase in MM incidence was 2.19% (95% CI 1.60 – 2.78, p < 0.001). Graphs in Fig. 1 show the trend of MM incidence from linear regression analysis, by gender: the upward trend was similar among the female (0.189 × 10^–5^ per year, *p* = 0.007) and the male population (0.113 × 10^–5^ per year, *p* < 0.001), with a slope 10 times steeper above the age of 65 (0.479 × 10^–5^ per year, *p* < 0.001) than below 65 (0.049 × 10^–5^ per year, *p* < 0.001) (analysis of covariance: F = 307.37, DF = 58; *p* < 0.001). Results from the univariate regression analysis were confirmed with Poisson regression analysis. The upward time trend was observed in the areas with incidence rate below the median (0.110 × 10^–5^ per year, *p* < 0.001), as well as in the areas where incidence rate was above the median (0.207 × 10^–5^ per year, *p* < 0.001), but its slope was twice as steep in the high incidence areas (analysis of covariance: F = 61.5, DF = 58; *p* < 0.001). To further validate our data base with cancer registry data, we contrasted the 1993–2002 data from the Sassari Cancer Registry to the same figures in our data base for the same area. In 1993–1997, the Sassari Cancer Registry data reported a MM incidence, based on the world standard population, of 2.7 × 10^–5^ among men and 2.1 among women^22^, which increased up to 3.5 among men and 2.9 in 1998–2002^23^. The corresponding figures for the same area in our data base were 2.4 for men and 1.7 for women in 1993–1997, and 2.8 for men and 2.1 for women in 1998–2002. In 2008–2012, MM incidence (world standard population) was stable for both genders in the northern part of the region (Sassari Cancer Registry: men 2.9 × 10^–5^; women 2.8 × 10^–5^), while it was 4.0 × 10^–5^ among men, more than double the 1974–2003 figure from the clinical database (1.8 × 10^–5^), and 2.1 × 10^–5^ among women, substantially similar to the 1974–2003 incidence (2.3 × 10^–5^), in the Nuoro Cancer Registry^2^.

### Geographic map of MM incidence

**Figure 2:**
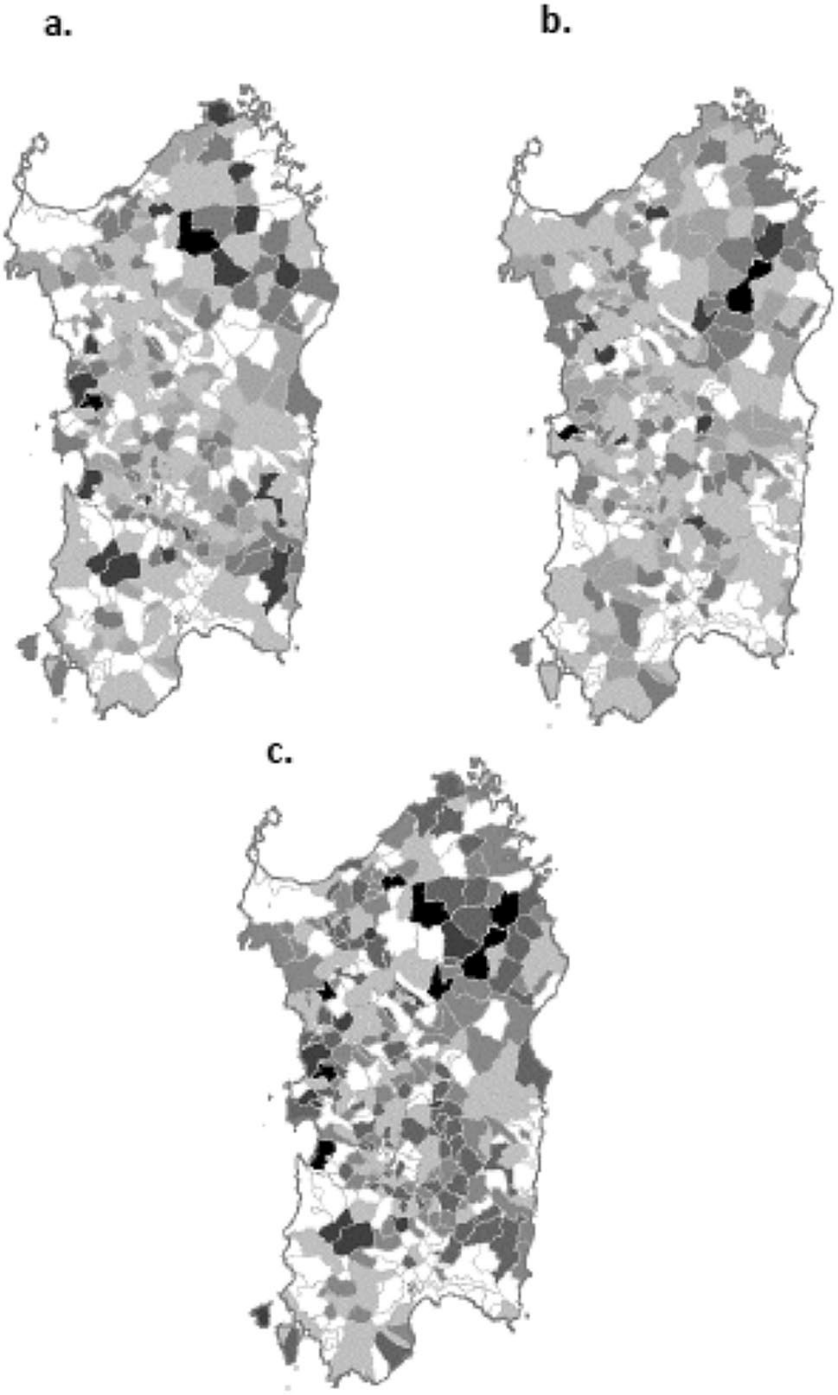
Map of multiple myeloma incidence in Sardinia, Italy. Likelihood ratios are presented by commune with the following colour scales: white < 0.165, pale grey 0.166–0.335, pale-medium grey 0.336–0.50, medium-dark grey 0.501–0.80, dark grey 0.801–0.95, black > 0.951. (**a**) males; (**b**) females; (**c**) total population. Maps of the administrative borders of the Italian communes were created by Spataro C and Piersoft Paolicelli F (https:\\umap.geonue.com/ en/map/confini-e-dati-statistici-dei-comuni-ditalia_297#8/40.102/8.973), using open access software by ANCITEL, a company providing services to the Association of the Italian Communes, under an ODBL (Open Database License) license. An ODBL license requires users to extend such license to the products created using it.

Figure 2 shows the map of the posterior probability (*P*) of a MM incidence rate above the critical rate in each of the 356 Sardinian communes, overall and by gender. Seven communes stand out exceeding the 95^th^ percentile distribution of the likelihood ratio. These are: Arborea (9 cases, likelihood ratio 20.2, *P* = 0.953), Padria (5 cases, likelihood ratio 20.2, *P* = 0.953), Benetutti (8 cases, likelihood ratio 46.8, *P* = 0.979), Bitti (11 cases, likelihood ratio 59.2, *P* = 0.983), Oschiri (11 cases, likelihood ratio 70.0, *P* = 0.986), Perfugas (10 cases, likelihood ratio 75.2, *P* = 0.987), and Seneghe (9 cases, likelihood ratio 177.9, *P* = 0.994). Another 25 communes have a posterior probability ranging 80–94%, based on 3 or more cases. The map shows a tendency of communes with a high posterior probability of an increased MM incidence to concentrate in the north-eastern area of the island, with the low probability areas located in the southern areas and in the two major urban areas, one, Cagliari, in the south, and the second, Sassari, in northwest Sardinia. Supplementary Table 2 shows the age and gender standardized incidence rates aggregated by health district.

### Analysis of MM risk factors

We explored with weighted multivariate regression analysis whether socioeconomic conditions, or size of livestock, or an aging population might have generated the wide geographic variability in MM incidence we observed. As an indicator of socioeconomic conditions, we used the Italian National Institute of Statistics (ISTAT) deprivation index, which combines the following: proportion of the resident population who attained elementary education at most; proportion of the resident population aged 15 years or older searching for an occupation; proportion of the active population engaged in manual work; proportion of rentals over the total residences; persons per room in the household^24^. Independent covariates were male/female ratio of the resident population, deprivation index, presence and size of livestock farms (cattle, sheep, and goats)^25^, and proportion of the population aged ≥ 65 years. We used the commune population sizes relative to the overall regional population as the weights. None of the covariates showed a significant association with MM incidence (Supplementary Table 3).

## Discussion

Our results show that in 1974–2003 MM incidence increased in both genders among the Sardinian population. Cancer Registry data confirmed such finding limited to the northern area of the region and the last decade covered by the database we used. There was no further increase in the subsequent years. The steeper slope of the regression line among the elderly suggests that the increasing aging of the Sardinian population and the increasing access of the elderly to specialized medical care over the study period might have contributed to a more frequent diagnosis of the disease along the years. However, the same upward time trend was also observed among the population aged < 65, indicating that other factors might have played a role, possibly interacting with genetic susceptibility to generate the steeper upward time trend we observed in high-risk areas.

Seven communes stand out with the highest probability of their rate exceeding that of the overall population of the region, four located in the north-eastern area (Benetutti, Bitti, Oschiri, and Perfugas) and three scattered in the north-west and central areas. These are mainly agricultural areas, six with archaeological remains indicating their origin dating back to prehistory, and one, Arborea, built in 1928 over a reclaiming land, previously covered by marshes in a malaria endemic area. Most of the population hosted in this new city came in the early years of its foundation from Veneto and Friuli, two regions in north-east Italy, and created a flourishing livestock and agricultural crop economy. This town is also known for the large size of its livestock, with two thirds of Sardinian cattle raised in its land. Although we are unaware of publications exploring the association between zoonoses and risk of multiple myeloma among livestock breeders, several papers investigated the association between risk of lymphoma and contact with livestock^26^. However, we did not find a relationship between presence of large cattle farms and MM incidence over the whole region. The analysis of cancer mortality and hospitalisation in areas at risk because of industrial or military settlements, reported an isolated excess of mortality from MM in an urban area in north-eastern Sardinia, and no excess in any of eight areas including major industrial settlements^27^. Our results confirm no excess of MM incidence in the communes surrounding the industrial areas in the Sardinian territory.

The finding of an excess risk associated with having a first-degree relative affected by MM, particularly among men, and African Americans, supports a role of genetic factors^28^. On the other hand, about 17% of MM heritability seems explained by the known gene variants^29^. Besides, based on results from molecular biology studies, aberrant class switch recombination occurring early in the natural history of MM suggests that environmental factors, such as high doses of ionizing radiation, and occupational exposure in the farming and petrochemical industries, might also contribute to increase risk^30^. The DNA damage resulting from environmental exposures would interact with the class switch recombination process to increase the risk of chromosomal translocations, oncogene deregulation, and malignant transformation^30^. In an analysis of MM risk related to occupation, a moderate increase in risk was reported in association with contact with livestock^31^. Also, gardeners and nursery workers combined, but not other farming jobs, metal processors, female cleaners, and occupations with high level exposure to organic solvents showed a moderate increase in risk^32^. Among lifestyle factors, a moderate alcohol intake might would reportedly convey protection^33^. We could not detect a role of deprivation index, an indicator of socioeconomic level, nor did the prevalence of elderly or the male/female ratio or the presence and size of livestock farming affect MM incidence. However, in our ecological analysis, the population of each commune was the unit, and not the individual. Unless the exposed represent a large proportion of the resident population and a strong association exists between the environmental exposure and the disease, the so-called ecological fallacy is likely to occur and to mask possible associations or to generate spurious increases in risk^34^.

We are not aware of genetic investigations aiming to identify the varying prevalence of gene polymorphisms implicated in MM among the Sardinian population. The small town of Arborea, with his peculiar modernist architecture, is home for about 4000 inhabitants, a large fraction of whom preserved their original language, diet, and habits. This population has a different ethnic origin than the rest of the Sardinian population, but it is unclear whether this might be related to the excess incidence of MM therein observed. Nonetheless, the incidence for the resident population (both genders), standardized based on the world population, was 4.6 × 10^–5^, 5.05 among men, and 4.2 among women. The corresponding rates in the Veneto region cancer registry were 4.5 for men and 3.7 for women in the IARC CI5 10th Edition^35^, and 4.1 for men and 2.7 for women in its 11th Edition^2^. The corresponding figures in the Friuli Cancer Registry were 3.8 for men and 2.9 for women^26^, and 3.3 for men and 2.5 for women^2^, consistent with what observed in the town of Arborea in 1974–2003.

The imprecise correspondence with Cancer Registry data might be due to the fact that the database we used includes the cases resulting from an exhaustive active search of the MM diagnoses in the registers of all clinical departments, followed by a double check of the clinical records of each case for the Durie and Salmon diagnostic criteria^36^ to come up with the diagnostic certainty of MM. These might not include all the incident cases reported to the Cancer Registries; besides, underdiagnosis of MM might have occurred among the elderly, particularly in the early years of creating the database we used. However, this would affect mostly small villages far apart from the specialized haematology units, located in the major urban centres; still, the elevated risk was mainly observed exactly in small towns, which would contrast this hypothesis.

For the same reason, post-diagnosis relocation of the families seems unlikely to have occurred. The exact address at the time of diagnosis was missing for 58/1606 patients (3.6%); it seems also unlikely that this might have affected the overall pattern.

An advantage of our study is that the diagnoses were all reviewed by the same expert haematologist (GB), thus preventing bias due to the varying diagnostic ability by time and geographic area and minimizing and spreading equally the probability of misdiagnosis over the whole region and along the study period. Although not exactly matching the Cancer Registry data limited to the overlapping period and to the northern part of the island for the reasons explained above, the similar figures calculated from our database confirm the completeness of its records.

## Conclusion

Our results describe for the first time increasing time trends of multiple myeloma over several decades among the population of the island of Sardinia. Multiple myeloma incidence increased in both genders, and particularly among the elderly and in high-risk areas. We also observed a clustering of high MM incidence in the north-eastern area, which might be of interest for future gene-environment interaction studies, with special focus on agricultural factors, such as use of pesticides, exposure to endotoxin, and contact with livestock and zoonotic agents. We could not identify a role of socioeconomic status, as indicated by the deprivation index, nor of livestock farming. Finally, our results might support the extension of the Cancer Registry coverage to the whole Sardinian population, which would be of paramount importance not only for fostering further research, but also for early detection of risk areas, so to promote effective preventive intervention, and for a rational planning of cancer treatment resources.

## Material and methods

A detailed description of the database of haemolymphatic malignancies we used in this study can be found elsewhere^20^. Briefly, it includes 14,744 incident cases of any haematological cancer, in both genders, and at any age diagnosed in the Italian region of Sardinia in 1974–2003. For the purposes of this analysis, we selected the 1606 MM cases, 781 males and 825 females.

For each commune (the smallest administrative unit in Italy), we calculated the total person-years for each gender and age group (0–24, 25–34, 35–44, 45–54, 55–64, 65–74, and 75 +) over the study period, from January 1974 through December 2003. The standardized incidence rate, annual and over the whole study period, of MM was calculated using the 1971, 1981, 1991, and 2001 census data of the regional population as the standard. Census figures were extended four years onwards and five years backwards to estimate the resident population in the intercensal years. The time trend along the study period was explored with the linear regression equation, and with Poisson regression, adjusting by age and gender. To compare results with those from the IARC CI5 volumes, we also standardized the regional, gender specific incidence rates using the standard world population. We used analysis of covariance to test the chance probability associated with the different slope of regression coefficients by gender, by age at diagnosis (below or above 65 years old), and by residence in an area with low vs high probability of MM occurrence, using the median as the cut point^37^.

The statistics to explore the spatial distribution of the probability of MM occurrence have been previously described in detail^38^. Briefly, we used a Bayesian approach, which allowed us to combine information on MM incidence over the island with that from the individual communes with the following equation:

1 $$P\left(\eta |d,I\right)=\frac{P\left(d|\eta ,I\right)P\left(\eta |I\right)}{P(d|I)}$$

where *P(η|d,I)* is the posterior probability distribution of MM incidence rate *η* for a given commune, after combining the data *d* for that commune with those from the whole region. $P\left(\eta |I\right)$ is the prior MM standardized incidence rate, η, from the background regional information; $P\left(d|\eta ,I\right)$ is the probability of getting $d$ for the commune assuming $\eta$ is true, and $P(d|I)$ sets to one the integral of the posterior probability *P(η|d,I)* over all possible values of $\eta$, so to obtain a probability density function.

To detect communes at high risk, we set the critical value of *p* = 0.001 in the prior probability distribution of MM incidence over the 356 Sardinian communes, and, for each commune and each gender and age subgroup, we calculated the likelihood ratio between the probability of a MM incidence rate higher than the critical probability level and that of a MM incidence rate consistent with what observed at the regional level.

Finally, we plotted on the regional map the probability associated with the likelihood ratio for each commune, using the following colour scale for the area of each commune, based on the quintiles of the probability distribution: white < 0.165, light grey 0.166–0.335, medium-light grey 0.336–0.50, medium-dark grey 0.501–0.80, dark grey 0.801–0.95. The few communes associated with a probability higher than 95% had the darkest black shade.

The methods used in this study do comply with the requirements for ecological studies, including the acknowledgement of the limitations of such study design, as highlighted by Rezaeian’s call for a still missing STROBE statement on ecological studies^39^. Our ecological study was based on the analysis of aggregated data; no humans were involved. Therefore, the informed consent requirement for participation does not apply. The use of these aggregated data for the purposes of scientific publication was approved by the Ethics Committee of the University Hospital of Cagliari (protocol N. PG 2019/18070, 18 December 2019).

## Supplementary Information

Supplementary Information.

## Data Availability

Data are preserved in the archives of the Department of Medical Sciences and Public Health of the Cagliari University in aggregated form, and they are publicly available as such. Please contact Prof. Pierluigi Cocco (http://pcocco@unica.it) for any request.
