# Author Correction: Time trend and Bayesian mapping of multiple myeloma incidence in Sardinia, Italy

**DOI:** 10.1038/s41598-022-07811-2

**Published:** 2022-03-02

**Authors:** Giorgio Broccia, Jonathan Carter, Cansu Ozsin-Ozler, Federico Meloni, Ilaria Pilia, Sara De Matteis, Pierluigi Cocco

**Affiliations:** 1 Department of Haematology and Bone Marrow Transplants, Hospital A. Businco, 09121 Cagliari, Italy; 2 University of Coventry, Coventry, CV1 5FB UK; 3 Department of Paediatric Dentistry, Hacettepe University, Faculty of Dentistry, Ankara, Turkey; 4 Department of Medical Sciences and Public Health, University of Cagliari, 09042 Monserrato, Italy; 5 Division of Population Health, Centre for Occupational and Environmental Health, University of Manchester, Manchester, UK

Correction to: *Scientific Reports* 10.1038/s41598-022-06745-z, published online 17 February 2022

The original version of this Article contained an error in the Results section, under subheading “Geographic map of MM incidence”,

“These are: Arborea (9 cases, likelihood ratio 20.2, *P* = 0.953), Padria (5 cases, likelihood ratio 20.2, *P* = 0.953), Benetutti (8 cases, likelihood ratio 46.8, *P* = 0.979), Bitti (11 cases, likelihood ratio 59.2, *P* = 0.983), Oschiri (5 cases, likelihood ratio 70.0, *P* = 0.986), Perfugas (10 cases, likelihood ratio 75.2, *P* = 0.987), and Seneghe (9 cases, likelihood ratio 177.9, *P* = 0.994).”

now reads:

“These are: Arborea (9 cases, likelihood ratio 20.2, *P* = 0.953), Padria (5 cases, likelihood ratio 20.2, *P* = 0.953), Benetutti (8 cases, likelihood ratio 46.8, *P* = 0.979), Bitti (11 cases, likelihood ratio 59.2, *P* = 0.983), Oschiri (11 cases, likelihood ratio 70.0, *P* = 0.986), Perfugas (10 cases, likelihood ratio 75.2, *P* = 0.987), and Seneghe (9 cases, likelihood ratio 177.9, *P* = 0.994).”

The original Article has been corrected.

