# Supplementary material for: Time trend and Bayesian mapping of multiple myeloma incidence in Sardinia, Italy
Source: Sci Rep. 2022 Feb 17;12:2736. doi: 10.1038/s41598-022-06745-z (PMC8854669; doi:10.1038/s41598-022-06745-z)
Supplement: Supplementary file 1 — Supplementary Information. [file 41598_2022_6745_MOESM1_ESM.docx]

Broccia G. et al.

Time trend and Bayesian mapping of multiple myeloma incidence in Sardinia, Italy.

**Supplementary material.**

**1. Statistical methods**

The Bayesian approach combines information about the incidence gathered from across the whole regional territory with specific data for individual communes. The key equation is:

$P\left( \eta|d,I \right)=\frac{P\left( d|\eta,I \right)P\left( \eta|I \right)}{P(d|I)}$ [1]

where P(η|d,I) refers to the posterior probability distribution of the cancer incidence rate η for an individual commune, hereafter referred to as incidence probability, i.e. the probability associated with each value of η, after the data d for the commune has been combined with data from the whole regional territory. $P\left( \eta|I \right)$ is the prior information about the standardized incidence rate, η, of childhood cancer given the background information, $I$, and is based solely on the regional data. $P\left( d|\eta,I \right)$ is the likelihood function, i.e. the probability of getting the measured data $d$ for the commune if we assume that the value of $\eta$ is true. The term $P(d|I)$ does not depend on η; it ensures that the integral of the posterior probability over all possible values of $\eta$ equals one, i.e. that $P\left( \eta|d,I \right)$ is a properly defined probability density function.

The prior summarises what is known about the standardized incidence rate, η, considering solely the regional data. In the definition, the following information is used:

- the incidence probability is a number in the interval between 0 and 1;
- the condition is rare, implying a low value for the incidence probability;
- it is acceptable to use data from the whole island when analysing data at the individual commune level;
- a few communes might have incidence probabilities higher than the rest of the island for genetic or geographic factors.

This information is summarized with a *β* distribution, where the mean is the same as the mean for the whole island, and the mode is half of the mean value. The exact shape of the prior probability density function changes with the age and gender subgroup being considered. The prior for an example can be seen in Figure 1; in this particular case, the mean is at 5.8 and the mode is at 2.9. Incident rates above 25 have an approximate probability of zero.

Whether an individual succumbs or not to the disease in a given year is a binomial process. Therefore, the number of cases observed $k$, in a population of size $n$, measured in person-years, is best described by a binomial distribution, which, since $k\ll n$, we can approximate with the Poisson distribution, which is

$P\left( d|\eta,I \right)=P\left( k,n|\eta,I \right)=e^{-n\eta}\frac{\left( n\eta\right)^{k}}{k!}$ [2]

where $n\eta$ is the expected number of cases in a population of size $n$. Given the number of cases and the population size in each commune, it is possible to calculate the probability of the observed number of cases for all values of $\eta$. Figure 1 shows the likelihood function for an example commune, which is a function of the incidence probability η. The mode is at 6.9 and there is a wide range of possible incidence rates, 0-35, which are consistent with the observed number of cases.

The posterior probability combines the prior knowledge with the information that can be obtained from the data via the likelihood function, according to Bayes rule, as shown in equation 1. Figure 1 shows the result. This analysis was repeated for the gender and age subgroups within each commune.

If we carry out the same type of analysis for the whole region, the posteriors are almost indistinguishable for any reasonable choice of prior. So, we used a uniform prior to generate the posterior function. Different priors at commune and regional levels are used because different information is available at the two levels. At the commune level we can use information that was derived at regional level. We then defined the critical value as the value such that the probability that the incidence rate for the region is less than the critical value is 0.999, i.e.

$$\text{Prob}\left( \eta<\eta_{c}|D,I \right)=\int_{0}^{\eta_{c}} P\left( \eta|D,I \right) \text{d}\eta=0.999$$

where $D$ is the data for the whole region. The data sets $d$ referred to earlier are for the communes and are not identical to the equivalent data $D$ for the region. The choice of 0.999 is arbitrary, but the subsequent analysis is not sensitive to the value chosen. In Figure 1, this value is marked by the thin vertical line. The critical values, as calculated at regional level, are given in Table 1.

We can now calculate the probability for the hypothesis $H1$, that the incidence rate in a commune is higher than the critical incident rate for the whole region:

$$\text{P}\left( H1|d,I \right)=\int_{\eta_{c}}^{1} P\left( \eta|d,I \right)\text{d}\eta$$

For the example in Figure 1 the probability of $H1$ is very small, as is true of most of the communes. This shows that for the example commune the probability that the incidence rate exceeds the critical rate is less than 0.01.

**Supplementary Figure 1.**

Plot of the prior probability distribution of the haematological malignancies in Sardinia, Italy, the likelihood function, and the posterior probability of a commune taken as an example. There is not a vertical scale to stress the relevance of the shape of the curve. The likelihood function has been rescaled. The thin vertical line indicates the critical value, and it shows that a substantial portion of the posterior probability curve lies beyond it.

**Supplementary Table 1.**

Critical thresholds $\eta_{c}$ used to calculate the likelihood ratio for each group in the fourth data set.

| Age and gender study group | Critical value of $\eta$ ($\eta_{c}$) |
| --- | --- |
| 35-44 Females | 0.0000155 |
| 45-54 Females | 0.0000410 |
| 55-64 Females | 0.0000920 |
| 65-74 Females | 0.0001675 |
| 75+ Females | 0.0001985 |
| 35-44 Males | 0.0000140 |
| 45-54 Males | 0.0000430 |
| 55-64 Males | 0.0001050 |
| 65-74 Males | 0.0001820 |
| 75+ Males | 0.0002540 |

**Supplementary Table 2.**

Incidence of multiple myeloma and number of cases by age groups (≤ 65 years, ≥ 66 years) by health district

| Health District | Geographic location* | Resident population | P-years | MM cases | Cases ≤ 65 years (%) | Cases ≥ 66 years (%) | sir** | 95% CI | sir *** | 95% CI |
| --- | --- | --- | --- | --- | --- | --- | --- | --- | --- | --- |
| Cagliari - west | 1 | 101,174 | 2,743,220 | 73 | 37 (51) | 36 (49) | 3.27 | 2.67 - 3.88 | 2.16 | 1.55 - 2.77 |
| Cagliari – Quartu S. Elena | 2 | 369,687 | 10,175,114 | 305 | 119 (39) | 186 (61) | 3.21 | 2.87 - 3.54 | 2.03 | 1.69 - 2.37 |
| Sulcis-Iglesiente | 3 | 126,134 | 4,069,458 | 109 | 43 (39) | 66 (61) | 2.72 | 2.21 - 3.22 | 1.82 | 1.32 - 2.32 |
| Sanluri - Guspini | 4 | 100,755 | 3,241,416 | 110 | 51 (46) | 59 (54) | 3.38 | 2.74 - 4.01 | 2.36 | 1.73 - 3.00 |
| Sarcidano - Trexenta | 5 | 43,634 | 1,438,192 | 52 | 15 (26) | 37 (74) | 3.18 | 2.20 - 4.16 | 2.12 | 1.14 - 3.10 |
| Sarrabus - Gerrei | 6 | 21,764 | 683,998 | 28 | 14 (50) | 14 (50) | 3.70 | 2.19 - 5.22 | 2.79 | 1.28 - 4.31 |
| Oristano - Ales | 7 | 116,895 | 3,676,866 | 134 | 35 (26) | 99 (74) | 3.36 | 2.75 - 3.98 | 2.01 | 1.40 - 2.63 |
| Lanusei - Tortolì | 8 | 55,689 | 1,784,574 | 40 | 20 (50) | 20 (50) | 2.21 | 1.51 - 2.90 | 1.60 | 0.90 - 2.29 |
| Bosa – Ghilarza - Macomer | 9 | 66,124 | 2,228,560 | 91 | 27 (30) | 64 (70) | 3.20 | 2.36 - 4.04 | 2.13 | 1.29 - 2.97 |
| Nuoro - Sorgono - SIniscola | 10 | 132,964 | 4,236,956 | 140 | 49 (35) | 91 (65) | 3.25 | 2.71 - 3.80 | 2.16 | 1.61 - 2.71 |
| Alghero | 11 | 72,503 | 2,331,596 | 77 | 25 (32) | 52 (68) | 2.87 | 2.13 - 3.61 | 1.86 | 1.13 - 2.60 |
| Ozieri | 12 | 32,166 | 1,073,848 | 36 | 15 (42) | 21 (58) | 2.89 | 1.80 - 3.99 | 1.98 | 0.88 - 3.07 |
| Sassari | 13 | 201,914 | 6,232,360 | 209 | 90 (43) | 119 (57) | 3.55 | 3.09 - 4.00 | 2.28 | 1.83 - 2.74 |
| Olbia - Tempio | 14 | 134,561 | 3,852,152 | 144 | 53 (37) | 91 (63) | 3.69 | 3.08 - 4.30 | 2.40 | 1.79 - 3.01 |

Notes:

* see the map in supplementary figure 2;

** age- and gender-standardized incidence rate x 10^-5^ (standard population: Sardinia 1971 – 2001);

*** age- and gender-standardized incidence rate x 10^-5^ (standard population: World standard population).

**Supplementary Figure 2**

Map of the Health districts in the region of Sardinia. To better represent geographical areas in a few instances 2-3 health districts have been combined.

**Supplementary Table 3.**

1974-2003 Multiple myeloma incidence in the 357 communes of the Italian region of Sardinia as a function of environmental and socio-economic covariates.

| Covariates | regression coefficient | standard error | p-value |  |
| --- | --- | --- | --- | --- |
| Deprivation index | -0.010 | 0.063 | 0.874 | |
| Male/female ratio in the resident population | 2.719 | 2.432 | 0.264 | |
| Proportion of elderly in the resident population | 0.011 | 0.020 | 0.586 | |
| Population size (relative to the regional population) | 0.051 | 0.028 | 0.068 | |
| Heads of cattle (100+ vs ≤ 99) | 0.271 | 0.269 | 0.315 | |
| Heads of sheep & goats (/1,000) | -0.007 | 0.005 | 0.143 | |
|  | R^2^ = 0.016 | | |  |
